# Bio-Inspired Superhydrophilic Urchin-Like Supramolecular Structures Formed by Co-Assembly of Simple Metabolites

**DOI:** 10.1021/acs.langmuir.6c03644

**Published:** 2026-10-20

**Authors:** Ruth Aizen, Om Shanker Tiwari, Gal Finkelstein-Zuta, Pini Shekhter, Davide Levy, Ehud Gazit

**Affiliations:** † The Shmunis School of Biomedicine and Cancer Research, The George S. Wise Faculty of Life Sciences, 26745 Tel Aviv University, Tel Aviv 6997801, Israel; ‡ Department of Chemistry, National Institute of Technology Warangal, Hanamkonda, Telangana 506004, India; § Jan Koum Center for Nanoscience and Nanotechnology, Tel Aviv University, Tel Aviv 6997801, Israel; ∥ Department of Materials Science and Engineering, The Iby and Aladar Fleischman Faculty of Engineering, Tel Aviv University, Tel Aviv 6997801, Israel

## Abstract

Numerous natural surfaces exhibit unique wetting behaviors, typically attributed to a synergy between two-tier micro/nanoscale hierarchical structures and specific material chemistry. Replicating these surfaces is often challenging because it relies on harsh, complex synthesis methods for inorganic materials. As a versatile extension of traditional self-assembly, heterogeneous molecular assembly offers a programmable route toward complex hierarchical biomimicry. Here, we study the coassembly of two simple metabolites, riboflavin and 2,6-diaminopurine, to demonstrate this approach for constructing functional materials. We found that the co-assembled structures exhibit an urchin-like morphology and employed various characterization methods to investigate the interaction between the two metabolite building blocks. The co-assembled structures exhibit superhydrophilic characteristics that were not achieved with unimolecular components, underscoring the advantages of a co-assembled system. Although the unimolecular components are hydrophilic and possess surface hydrophilic groups, as demonstrated by X-ray photoelectron spectroscopy (XPS) analysis, this alone is insufficient to construct a superhydrophilic surface. This was confirmed by measuring the water contact angle (WCA) of the coated hydrophilic glass coverslip in contact with the co-assembled structures and the unimolecular components, which rendered the glass superhydrophilic. Inspired by the amphiphilic nature of the Namib desert beetle’s back used for water harvesting, we coated a hydrophobic surface with the co-assembled structures. This modification transformed the surface to superhydrophilic, highlighting the importance of surface texture in conjunction with surface chemistry in establishing superwetting behavior. This study provides important insights into the interplay between the coassembly of metabolite building blocks and functional materials and establishes an innovative yet simple design route for developing functional surfaces.

## Graphical abstract

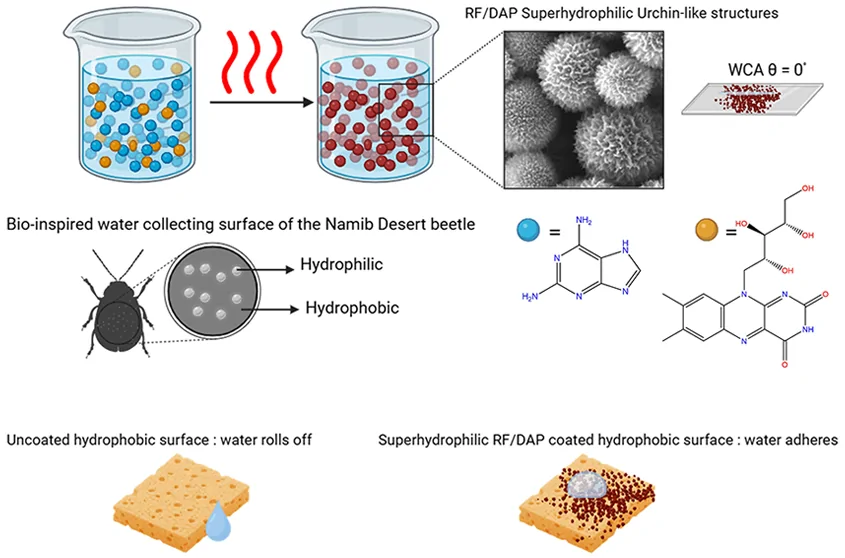

## 1 Introduction

Various life forms in nature display a high level of adaptability to their environments through the use of sophisticated material interfaces.^1–3^ This is exemplified by numerous biological systems, such as the self-cleaning of lotus leaves,^4,5^ the water-walking abilities of water striders,^6^ the ultraslipperiness of pitcher plants,^7,8^ the directional liquid adhesion of butterfly wings,^9,10^ the water collection capabilities of beetles,^11^ and more. The versatile interactions of these natural surfaces with fluids, or special wettability, are enabled by their unique micro/nanoscale surface structures and intrinsic material properties.^1,12,13^ The lotus leaf, for instance, possesses microscale wax structures combined with nanostructures that create a superhydrophobic surface,^4,5^ causing water droplets to bead up and roll off, carrying dirt and remains with them. In contrast, the leaves of Nepenthes pitcher plants exhibit a hydrophilic, slippery peristome (rim) characterized by an intricate micropattern of parallel ridges and grooves. These characteristics promote strong adhesion to water, facilitating the trapping and digestion of insects.^3,7,8^ Inspired by nature, many research efforts have been made aiming to mimic these features and functionalities for various applications, such as self-cleaning surfaces,^14^ water harvesting,^15–18^ antifogging materials^19^ and more. The construction of these materials often requires synthesis methods that usually employ harsh and complicated procedures.^14,18,20^ These include chemical vapor deposition (CVD),^21^ lithography,^22^ 3D printing,^23^ electrospinning^24^ and plasma treatment,^25^ which may hamper the use and production of these materials. The self-assembly of small organic molecules, specifically of natural metabolites, presents a promising avenue for constructing structures with complex functionalities and versatile physical and chemical properties.^26–30^ This approach offers inherent advantages such as biocompatibility, biodegradability, and cost-effective fabrication. Nonetheless, constructing functional supramolecular structures by unimolecular assembly processes can often be hampered by a lack of chemical diversity and functional complexity.^31^ Supramolecular coassembly has emerged as a promising avenue to overcome these limitations, facilitating the cooperative integration of diverse functional building blocks. The noncovalent interactions within the supramolecular coassembly enable simple fabrication and avoid complex synthesis, purification, and processing compared to covalent copolymers. The combination of different building blocks synergistically combines their properties, thereby producing new and different functions arising from a co-assembled system, which are not possible using a single component self-assembly process. Moreover, adjusting the ratios of the individual building blocks allows harnessing the adaptable coassembly mechanism to tune the morphology and the resulting physical, chemical, and mechanical properties.^32–36^

Here, we focused on the metabolite riboflavin (RF), which is primarily recognized for its role as an essential water-soluble vitamin and is involved in various metabolic pathways within the cell.^27^ Interestingly, it has also been found to form optically functional supramolecular structures within the tapetum lucidum of lemurs and cats.^37,38^ The tapetum is a reflecting layer found behind the retina and is responsible for enhanced night vision in low-light settings.^39^ Riboflavin is T-shaped, incorporating three fused heterocyclic rings, an isoalloxazine ring and a tetrahydroxypentanal side chain. The isoalloxazine has a fused thymine-like structure, highlighted in Scheme 1a. These molecular attributes motivated us to test the coassembly of riboflavin (RF) together with different nucleobases, relying on the specificity of the hydrogen-bonding interactions between complementary base pairs.

**Scheme 1:**
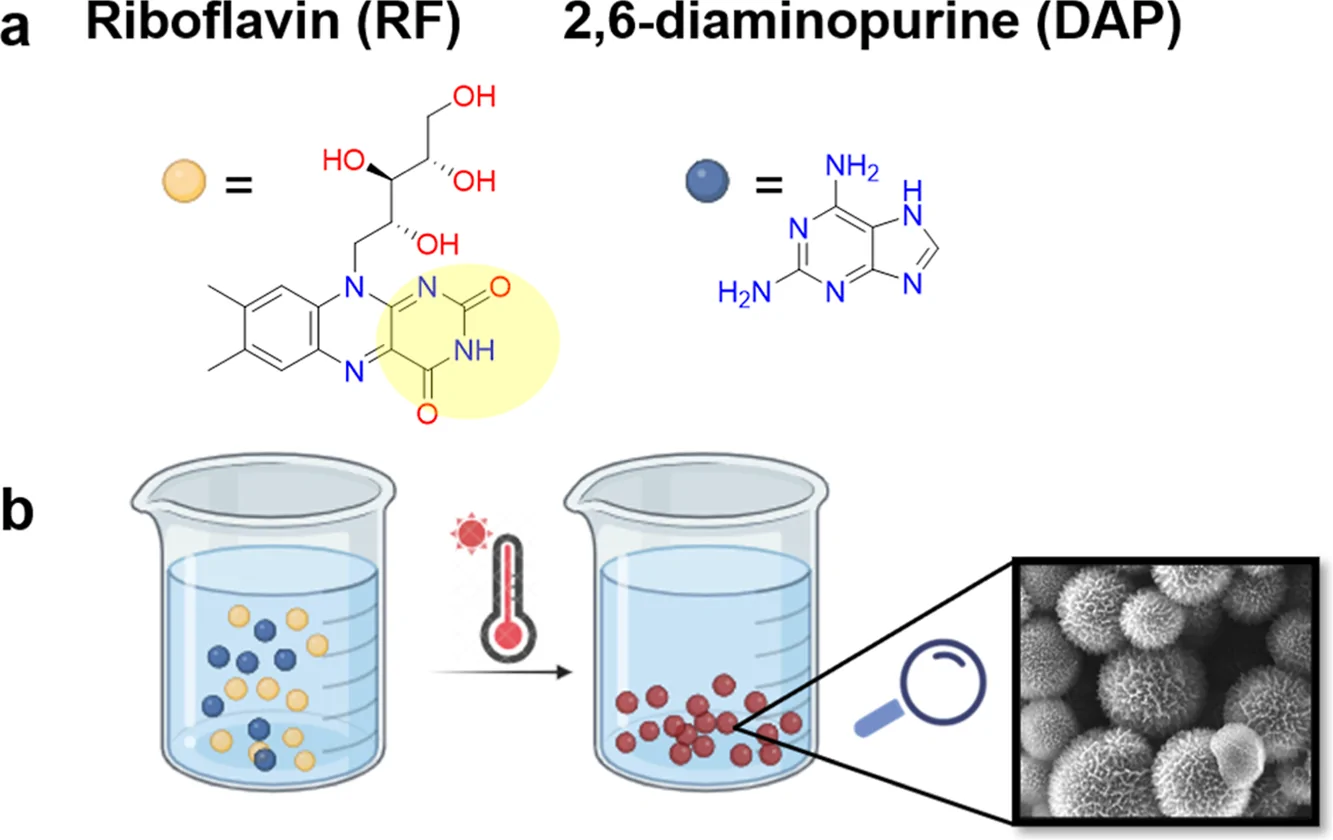
(a) Molecular Structure of RF and DAP; Blue and Yellow Balls Represent DAP and RF, Respectively; (b) Schematic Illustration of the Process of the Coassembly Between RF and DAP

## 2 Results and Discussion

To test our hypothesis, we screened the coassembly of RF with different nucleobases that potentially can form hydrogen bonds with the thymine-fused ring of RF. Specifically interesting is 2,6-Diaminopurine (DAP, Scheme 1a). DAP is an adenine analogue found in *Siphoviridae* viruses,^40^ containing an additional amine group at the 2-position on the purine ring. It can potentially form three hydrogen bonds with the isoalloxazine ring of RF (Scheme 1a). Coassembly was achieved by mixing 1,1,1,3,3,3-hexafluoro-2-propanol (HFIP)-dissolved RF with water-dissolved DAP at a 1:1 molar ratio, followed by heating to 90 °C and overnight incubation (Scheme 1b). This process resulted in the appearance of small red precipitates. Adjusting the molar ratio by reducing the concentration of RF in the solution resulted in a color change of the precipitates (Figure 1a–d inset). Further analysis using Scanning Electron Microscopy (SEM) revealed that the precipitates showed an urchin-like morphology, with microspikes/hairs (Figures 1a–d and S1). Figure 1e and f show the morphology of the unimolecular assemblies of DAP and RF, respectively, using the molar concentration at a 1:1 molar ratio of RF and DAP. Here, it is important to note that since RF was introduced from an HFIP stock solution, decreasing the amount of RF also decreased the final HFIP content; therefore, the observed morphological differences may reflect contributions from both composition and solvent environment. More specifically, to promote the formation of coassembly, RF dissolved in HFIP is added to an aqueous DAP solution at 90 °C, resulting in simultaneous RF-DAP association and HFIP dilution. This change in the solvent environment may influence local supersaturation, nucleation, and subsequent supramolecular growth. Therefore, although the observed urchin-like morphology is associated with RF-DAP coassembly, kinetic factors such as solvent exchange, order of addition, cooling, and aging also contribute to the final hierarchical structure.

**Figure 1:**
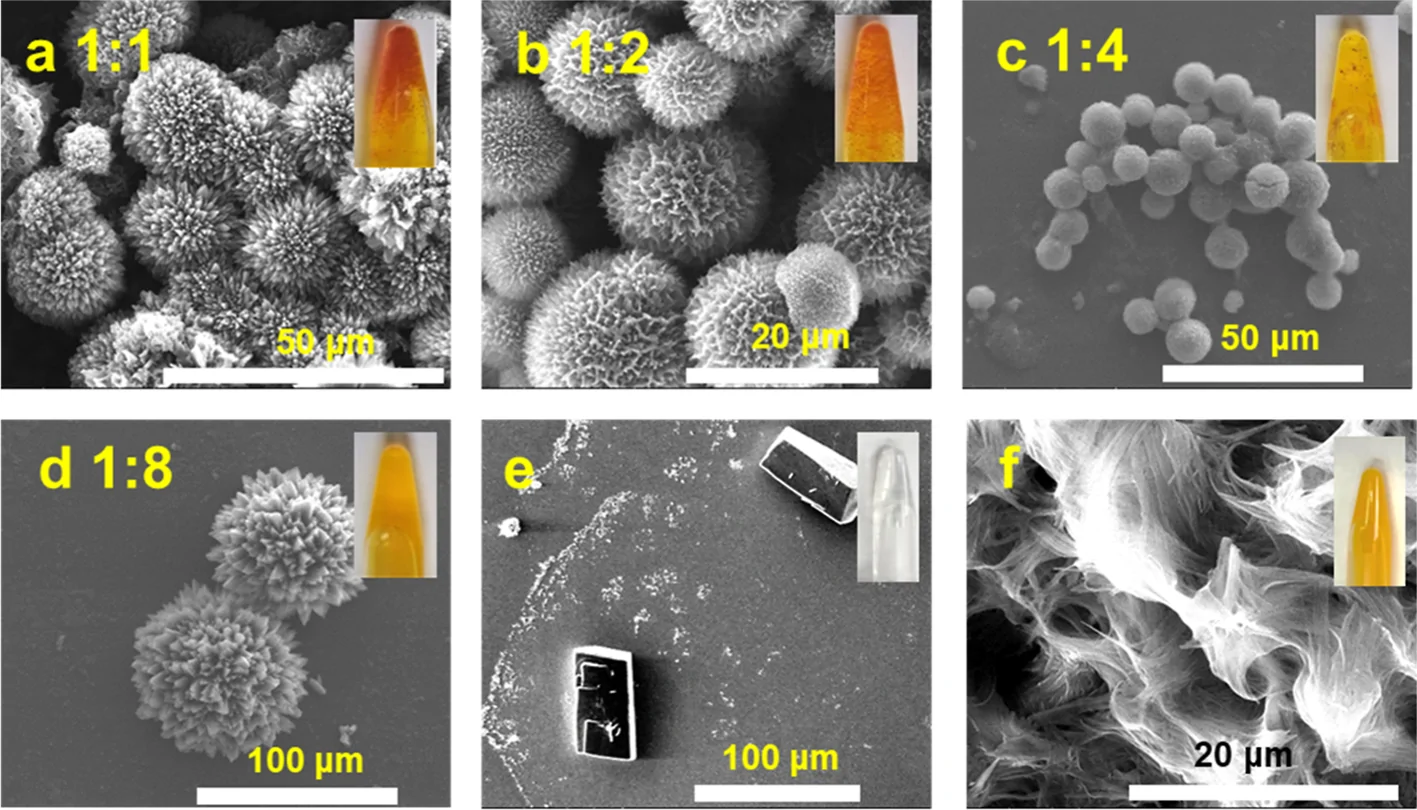
(a–d) SEM images of RF and DAP coassemblies at different molar ratios, (a) 1:1, (b) 1:2, (c) 1:4, and (d) 1:8. The inset shows images of the visual appearance of the corresponding coassemblies or the individual building blocks (1:1). (e,f) SEM images of the unimolecular components, (e) DAP and (f) RF.

The morphology of the RF-DAP assemblies showed a clear dependence on the RF-DAP ratio (Figure 1). SEM images indicate that the morphology and apparent size of the RF-DAP assemblies vary with composition, with DAP-rich conditions producing fewer structures (Figure 1c,d inset). At RF-DAP ratios of 1:1 and 1:2, well-developed urchin-like structures with pronounced radial surface features were observed. Increasing the relative amount of DAP to 1:4 resulted in smaller, more rounded structures with less pronounced radial features, indicating a change in the growth of the co-assembled structures. These morphological changes suggest that the relative concentration of RF and DAP affects both the formation and subsequent growth of the coassemblies. In contrast, neither DAP nor RF alone produced the characteristic urchin-like morphology, supporting the role of RF-DAP coassembly in directing the observed architecture. Interestingly, when trying to replace DAP with other nucleobases such as adenine (A), uracil (U), cytosine (C), or thymine (T) and mixing with RF at a 1:1 ratio, the color change and the resultant urchin-like structure were not evident (Figure S2). The selective formation of the urchin-like morphology with DAP cannot be attributed simply to its ability to form hydrogen bonds, as the other nucleobases tested also contain hydrogen-bond donor and acceptor groups. However, DAP differs from adenine by an additional exocyclic amino group at the 2-position, which changes the number and spatial arrangement of its hydrogen-bonding sites. DAP is known to form three hydrogen bonds with thymine- and uracil-type groups. Since the isoalloxazine ring of RF contains a thymine-like structure, this additional amino group may allow DAP to interact with RF in a different and potentially more favorable geometry. Thus, the selective formation of the RF-DAP urchin-like structures may arise not simply from the number of hydrogen bonds, but from the specific geometry and complementarity of the interactions between RF and DAP, which may alter molecular packing and direct the subsequent supramolecular growth. Since mixing RF with DAP at molar ratios of 1:4 and 1:8 yielded a very small amount of precipitation, we carried on our study of the co-assembled structures using the 1:1 and 1:2 molar ratios. To confirm that coassembly indeed took place, we examined the structures formed by RF and DAP using Time-of-Flight Secondary Ion Mass Spectrometry (ToF-SIMS) analysis (Figure 2). This method can be employed to identify the presence of two different molecules in a preformed nanostructure prepared in solution by the coassembly of two monomers.^41^ Chemical ion mapping was applied by selecting specific ions from the positive mass spectra to observe their location over a precise area (Figure 2b and d). This was achieved by separately fixing the red color for mass 151 *m*/*z* (DAP) and the green color for ions corresponding to mass 377 *m*/*z* (RF). Furthermore, the coassembly of these monomers was validated by overlapping the red and green channels. As observed in the far-right image of Figure 2a and c, the merged region demonstrating distinctive, yellow-colored dots arises due to the coassembly of RF and DAP.

**Figure 2:**
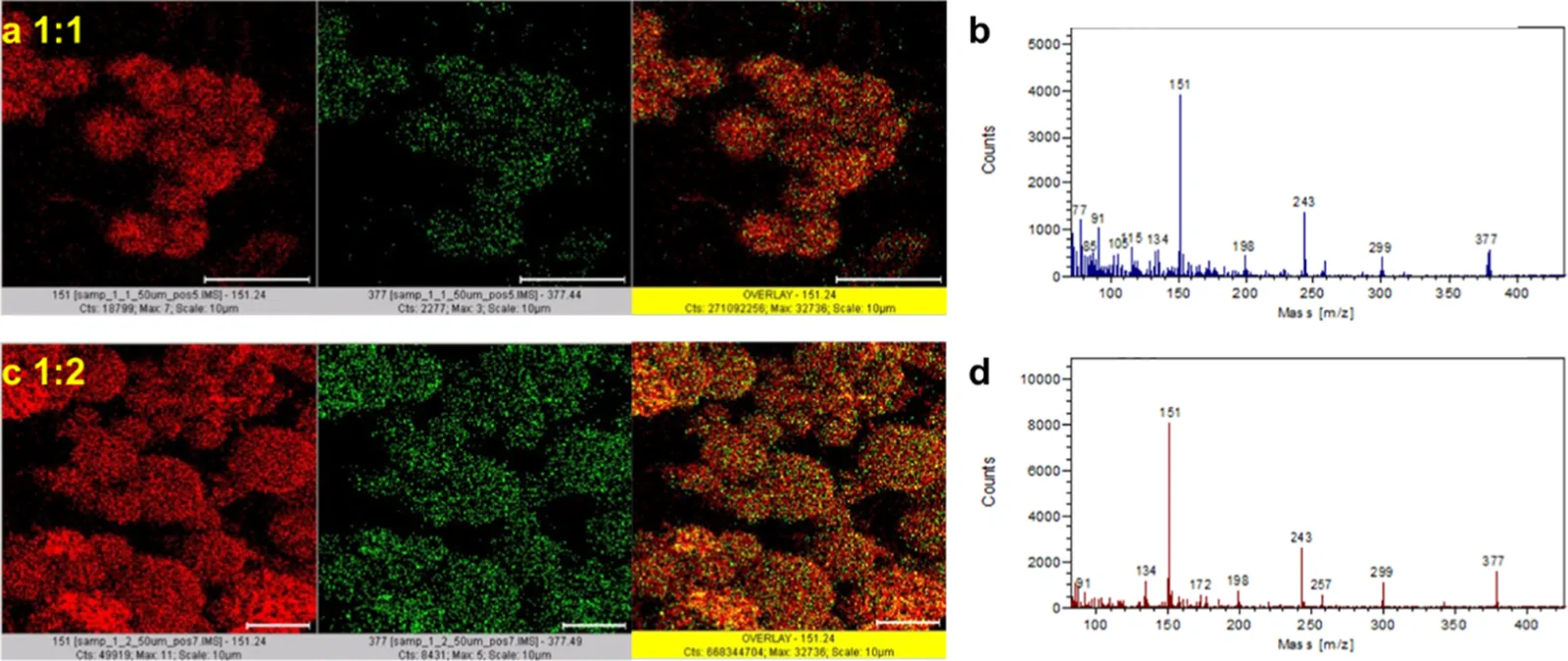
ToF-SIMS analysis of the chemical composition of the coassemblies. (a,c) ToF-SIMS chemical maps of DAP (left), RF (middle), overlay (right) at 1:1 and 1:2 molar ratios, respectively. (b,d) Mass spectra of 1:1 and 1:2 molar ratios, respectively, obtained from the ToF-SIMS.

The co-assembled structures (at both molar ratios) were also examined by Powder X-ray Diffraction (PXRD). The PXRD patterns of the unimolecular components and of the co-assembled structures were recorded. While RF and DAP both exhibited strong, sharp peaks indicating a degree of crystallinity in the unimolecular structures, the co-assembled urchin-like structures appeared to have only a few distinctive peaks and did not overlap with the peaks presented by either RF or DAP (Figure 3a,b). This further confirms that the coassembly presents a new structure different from that of a single RF or DAP.

**Figure 3:**
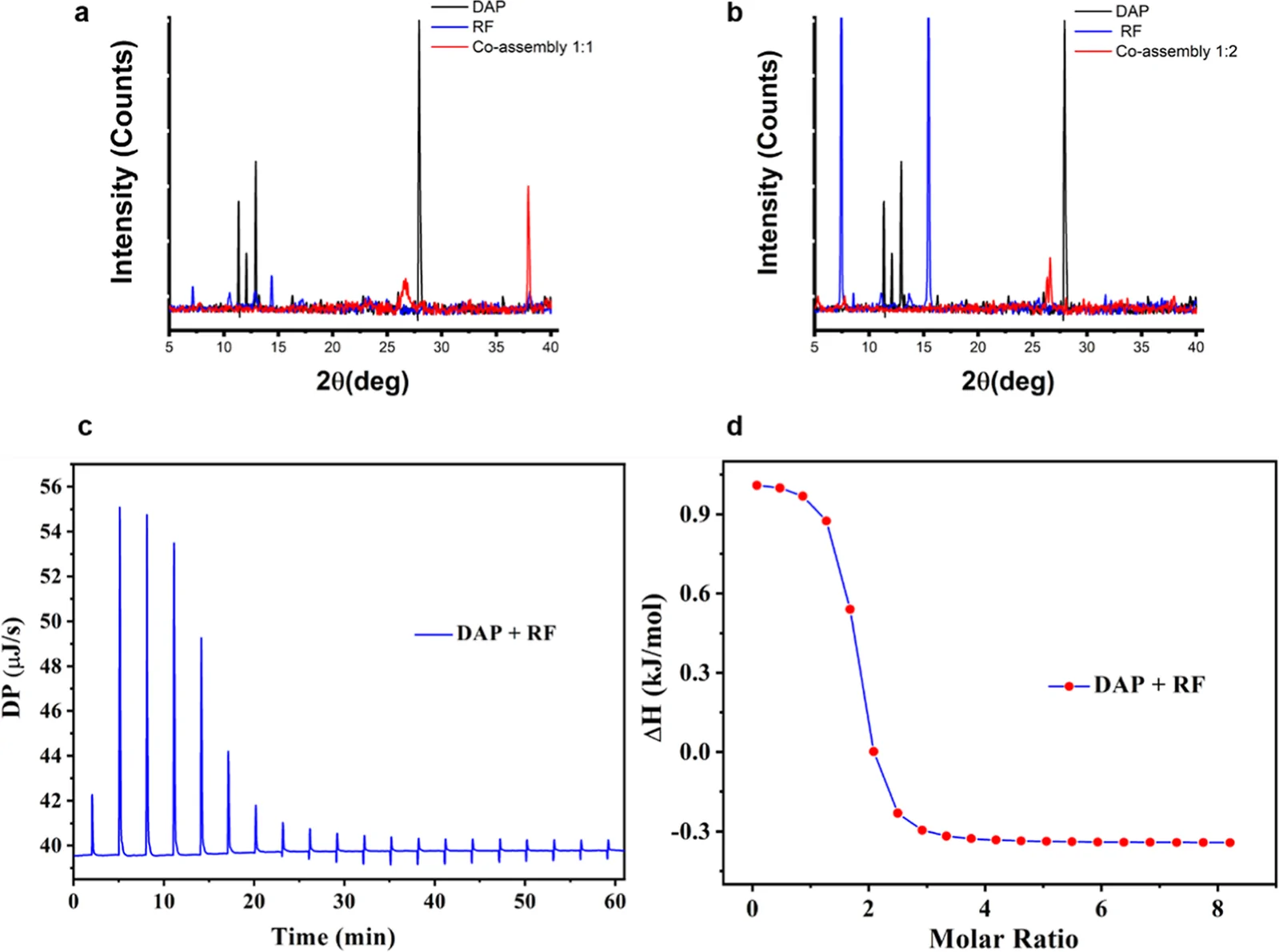
PXRD analysis of the co-assembled structures and the unimolecular components, RF and DAP, at a molar ratio of (a) 1:1 and (b) 1:2. (c) Heat pattern during DAP and RF binding. (d) The enthalpy changes upon increasing the DAP molar fraction.

To evaluate the thermodynamic properties of RF interaction with DAP, Isothermal Titration Calorimetry (ITC) measurements were performed. A freshly prepared solution of DAP was titrated into a cell containing RF to measure the corrected heat and enthalpy values. Results of the titration profile and the thermodynamic values were calculated and are displayed in Figure 3c and d. The calculated constants are presented in Table S1. Titration of DAP with RF resulted in endothermic peaks (Figure 3c). The calculated dissociation constants (K_d_) confirmed that RF interacted with DAP, exhibiting moderate association (27.1 × 10^–6^ M and Δ*G* = −26.1 kJ/mol). Interestingly, the endothermic pattern suggested that the interaction was driven by entropy.^33,34^ This is indicated by the calculated values of Gibbs free energy, where Δ*G* < 0 due to the relation *T*Δ*S* > Δ*H*. It was previously reported that entropy–driven interactions occurred primarily via hydrophobic contacts, whereas enthalpy-driven binding occurred via hydrogen bonding and electrostatic interactions.^42^ Thus, the ITC results may imply that the interactions of RF and DAP in this experiment occurred mainly through hydrophobic interactions. Presumably, the π-stacking of the aromatic rings of RF and the conjugated ring of DAP contribute significantly to the interaction.

Importantly, the ITC measurements were performed in 1× PBS at 25 °C and therefore do not directly describe the thermodynamics of the RF-DAP assembly process carried out in HFIP/water at 90 °C. Instead, the ITC data provide evidence for RF-DAP association under controlled aqueous solution conditions. The thermodynamic parameters prove to be supportive of molecular association rather than a direct thermodynamic description of the complete urchin formation process. The ITC measurements further support the interaction between RF and DAP, with an apparent stoichiometry of *N* = 1.64. The noninteger stoichiometry suggests that the interaction may be more complex than a simple 1:1 association; however, the ITC data alone does not allow the assignment of specific RF: DAP binding configurations. Further structural studies, including 2D NMR and solid-state NMR, could provide additional information on the molecular interactions and organization within the RF-DAP coassembly. Since the 1:1 molar ratio structure was much more challenging to handle (they adhere strongly to the Eppendorf tubes and tips), we next continued our study using only the 1:2 co-assembled structure. To investigate the changes that occur during the coassembly process, we measured the UV–vis spectra of the unimolecular components RF and DAP and of the coassembly in the solution state, immediately after the addition of RF to the DAP solution (Figure 4a). Both the coassembly and RF solutions gave rise to two peaks in the UV–visible spectrum, while DAP showed no peak in this region. The peak of RF at 446 nm was due to the π–π* transition of RF. In the coassembly, the peak was red-shifted to 462 nm, indicating that the π* electrons were stabilized through π–π stacking interactions during the assembly process. The peak at 369 nm was due to the π–π* transition and *n*–π* transition of RF, and this peak also showed a redshift to 379 nm, indicating that the π* electrons had been stabilized during the coassembly process.^41,43,44^ To gain further insight into the molecular organization of the co-assembled structures, Fourier Transform Infrared Spectroscopy (FTIR) was applied to analyze the RF and DAP self-assemblies and the coassembly (Figure 4b,c). The stretching frequency of RF was detected at 3405 cm^–1^, corresponding to the alcoholic –OH group of the ribityl chain; this peak was shifted to 3450 cm^–1^ in the coassembly, indicative of H-bond formation (Figure 4b). The –NH_2_ and –NH stretching frequencies of DAP appeared at 3355 cm^–1^ (symmetrical stretching) and 3176 cm^–1^ (asymmetrical stretching). In the coassembly, these peak intensities and positions were either reduced or shifted to lower frequencies, supporting the involvement of these groups in the formation of H-bonds between RF and DAP (Figure 4b). Also, the aromatic C=C stretching and C=N stretching modes of RF appeared at 1537 and 1653 cm^–1^, respectively. In DAP, the C=C and C=N stretching modes appeared at 1594 and 1622 cm^–1^, respectively. However, in the coassembly, we observed only one broad peak at 1655 cm^–1^, which may indicate that these two molecules form a strong noncovalent (π–π) interaction during the assembly process (Figure 4c). The peak of RF at 1720 cm^–1^ may be ascribed to the C=O stretching frequency (Figure 4c). This peak seems to disappear in the coassembly, probably indicating that the C=O group of RF is involved in significant hydrogen bonds in the assembly.^41,45^ Therefore, we can infer that although hydrophobic interactions are predominant in the formation of the structures, hydrogen bonds also contribute to the overall assembly process.

**Figure 4:**
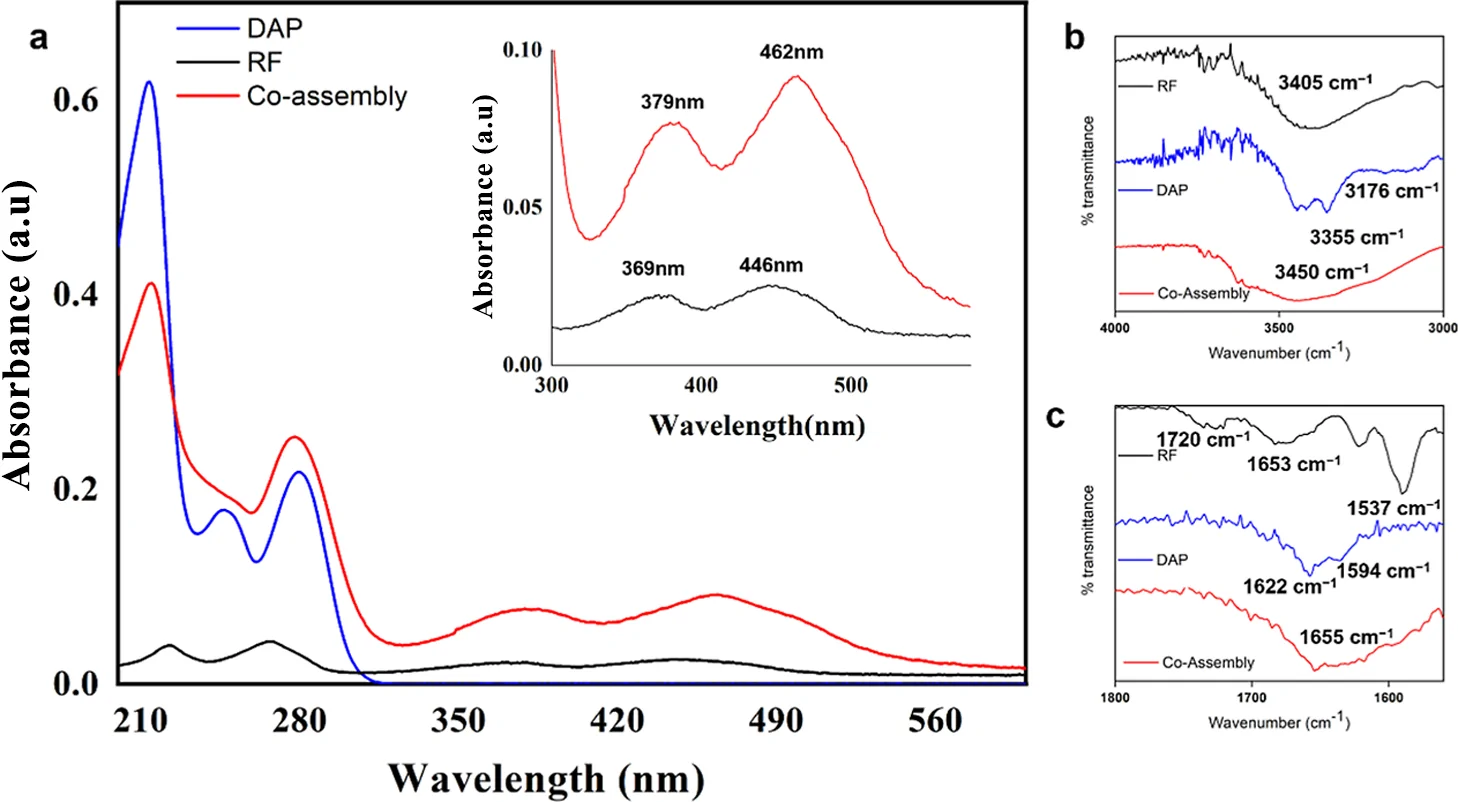
(a) UV–vis spectra of RF, DAP, and the coassembly in solution. The inset shows a zoom-in of the UV–vis spectra between 300 and 600 nm. (b,c) FTIR analysis of RF, DAP, and the coassembly at different wavelength ranges of the spectrum.

Thus, the spectroscopic results indicate that the incorporation of DAP changes the intermolecular interactions and molecular organization of RF. The changes observed in the amino groups of DAP and the hydroxyl and carbonyl groups of RF by FTIR support their involvement in hydrogen-bonding interactions during coassembly. In addition, the shifts in the UV–vis absorption bands of RF indicate a change in the electronic environment of the isoalloxazine chromophore upon coassembly, consistent with changes in aromatic interactions between the molecules. Together with the distinct PXRD pattern of the co-assembled structures and the colocalization of RF and DAP observed by ToF-SIMS, these results support the formation of a new RF-DAP molecular organization.

Moreover, previous structural studies of riboflavin-purine complexes have shown that hydrogen bonding involving the isoalloxazine moiety can occur simultaneously with π–π interactions between the aromatic rings. In particular, riboflavin-adenine complexes exhibit hydrogen bonding between adenine and the thymine-like region of the isoalloxazine ring together with alternating stacking of the adenine and isoalloxazine rings.^46,47^ As mentioned before, DAP possesses an additional amino group compared with adenine, which provides an additional hydrogen-bond donor and may alter the geometry and cooperativity of its interaction with RF. We therefore propose that the combination of hydrogen bonding and aromatic interactions between RF and DAP modifies the molecular packing and consequently the directionality of supramolecular growth. Rather than propagating into the extended sheet-like structures observed for RF self-assembly, the RF-DAP system may form anisotropic substructures that subsequently organize radially from nucleation centers. Radial morphologies in supramolecular and crystalline systems are commonly associated with anisotropic growth accompanied by branching and/or secondary nucleation.^48,49^ Thus, we suggest that RF-DAP molecular recognition alters the packing and growth pathway of the assembly, while subsequent radial growth and branching give rise to the observed urchin-like architecture. The present measurements do not directly resolve the molecular packing within the urchin-like structures, and therefore the precise molecular mechanism remains to be established. Future molecular modeling, including DFT calculations and molecular dynamics simulations, could provide further insight into the preferred RF-DAP binding configurations and the relative contributions of hydrogen bonding and π–π interactions to the molecular organization.

Typically, self-assembled structures with urchin-like or flower-like morphology are largely composed of inorganic oxides, often showcasing superhydrophobic properties attributed to their microstructure.^50^ The presence of nanoscale surface roughness and hierarchical structures creates air pockets or “lotus leaf-like” structures, further reducing the contact area between water and the surface.^1–4,12,13^ However, our results revealed an opposite observation. When depositing the co-assembled structures onto a hydrophilic glass coverslip and measuring the water contact angle (WCA) of the coverslip (θ = 57 ± 0.1°), compared to the coated coverslip, we discovered that the coated glass coverslip became superhydrophilic with θ = 0°. Water molecules spread out and wet the surface completely. Figure 5a shows the contact angle of a plain glass coverslip, and Figure 5b shows three consecutive images taken after the water drop encountered the coated glass coverslip.

**Figure 5:**
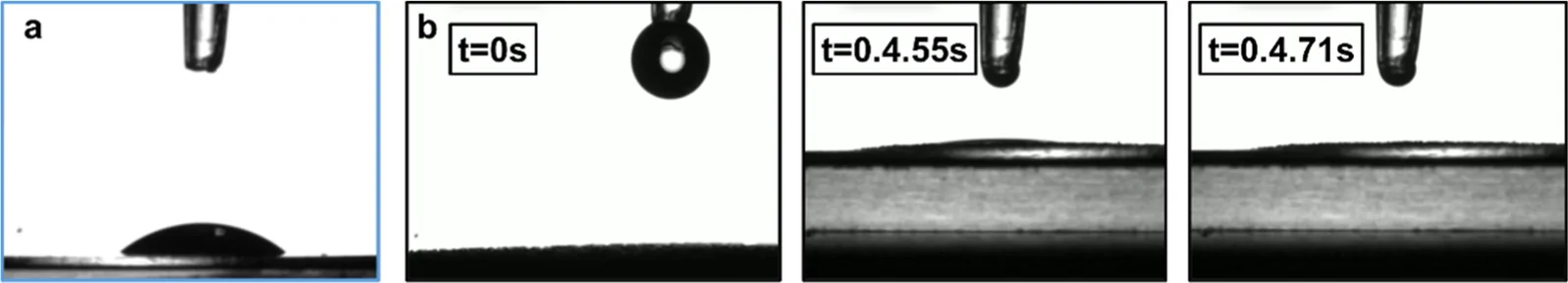
Photographs of the WCA of (a) a glass coverslip, (b) a glass coverslip coated with the co-assembled structures. The three consecutive photographs were taken before the water drop contacted the surface (*t* = 0) and immediately after contact *t* = 0.4.55 s. Complete spreading of the water drop occurred within 0.4.71 s.

Superhydrophilicity is usually achieved by a combination of sufficient roughness on the material surfaces and hydrophilic chemical compositions of the material.^1,12,13^ Based on the SEM images shown in Figure 1, the structures exhibit an urchin-like structure with microspikes generating hierarchical roughness. To assess the surface hydrophilicity of the co-assembled structures, we used X-ray photoelectron spectrometry (XPS). XPS is a surface-sensitive analytical technique that provides information about the elemental composition and chemical states of the top few nanometers of a material’s surface.^51^ As shown in Figure 6a,b, the N 1s spectra of the co-assembled structures have signals at ∼397.9–398.2 eV, 399.0–399.4 eV and 400.0–400.5 eV belonging to the C=N–C, the C–NH_2_, and the N–C=O, respectively. This suggests that those groups dominate the surface of the co-assembled structures. Also, compared with the XPS spectrum of RF, the (N + O)/C element ratio of the co-assembled structures increased from 60 to 66% (Figure 6c). Although the increment is not extremely high, it probably still contributes to the superhydrophilicity presented by the co-assembled structures. From these results, we can infer that the superhydrophilicity observed is indeed a combination of surface chemistry owing to the hydrophilic amines and carbonyl groups and the urchin-like spiky microstructure, which adds to surface roughness.

**Figure 6:**
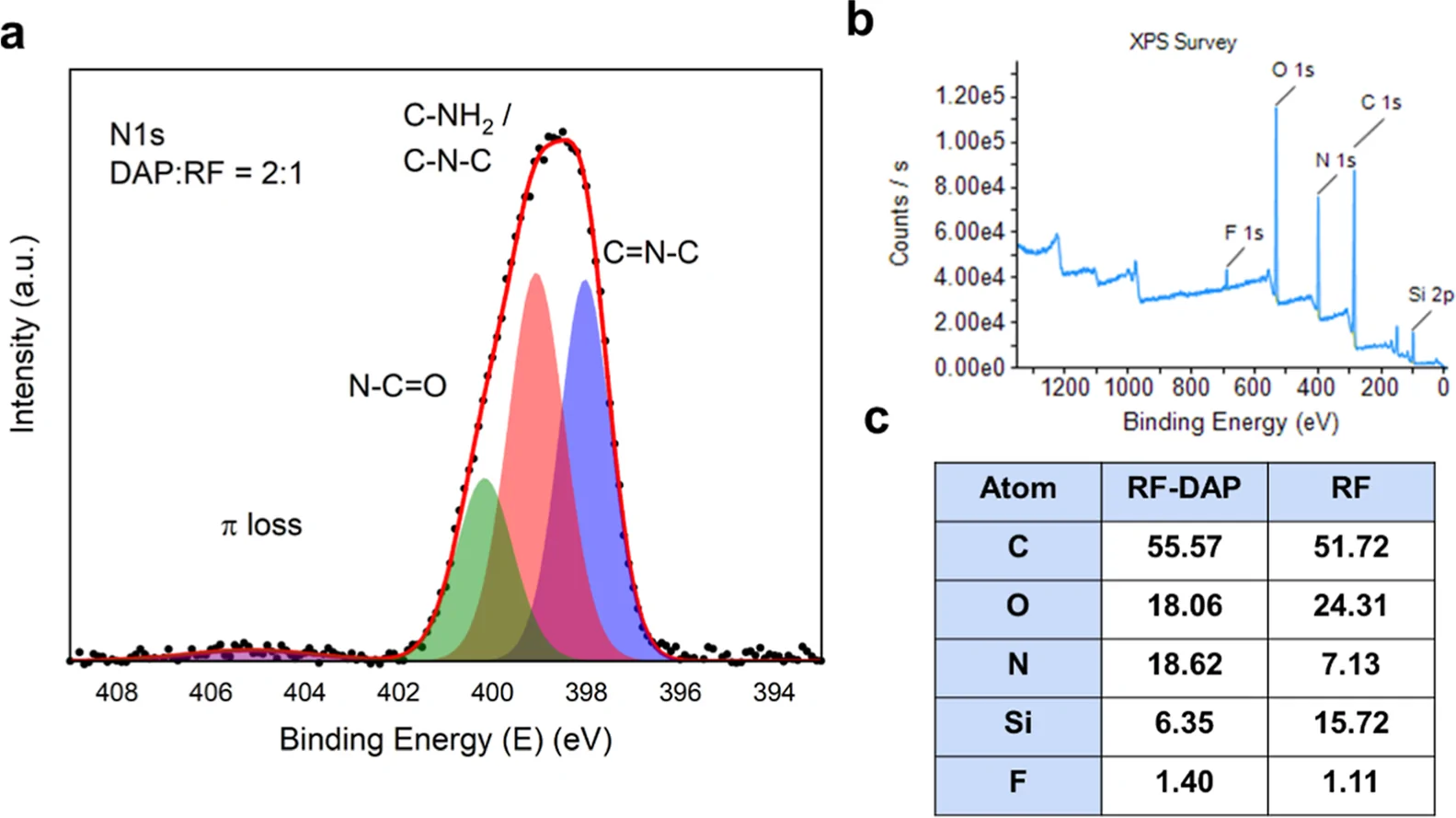
(a) High-resolution N 1s XPS spectra of the co-assembled structures. (b) XPS spectrum of the co-assembled structures. (c) Atomic concentration of the co-assembled structures compared to RF.

This combination of chemical heterogeneity and surface topography to achieve unique wettability is inspired by nature. For example, the Namib Desert beetle collects drinking water from fog on its back.^1,11^ The beetle’s back consists of an array of surface bumps decorated on top with hydrophilic spots (∼100 μm in diameter) on a superhydrophobic wax region. The small water droplets in the morning fog accumulate on the hydrophilic area and coalesce. When the weight of a growing droplet is sufficient to overcome the binding forces of the hydrophilic region, it detaches and rolls down the superhydrophobic surface to the beetle’s mouth. This combination of hydrophilicity–hydrophobicity (amphiphilic surface) inspired us to attempt to coat a hydrophobic surface and examine its wetting properties. For this aim, the co-assembled structures were deposited on a hydrophobic porous Cu foam. A notable transformation occurred in the WCA of the coated Cu foam, decreasing from θ = 126 ± 0.1° to θ = 0° (Figure 7a,b, respectively). This result indicates that the surface properties have changed from hydrophobic to superhydrophilic. Notably, when depositing DAP on the surface of the Cu foam, the WCA was only θ = 52 ± 0.1°, Figure S3 (RF did not adhere well to the surface and was dissolved and washed away with water). These results clearly demonstrate the advantages of the co-assembled urchin-like structure compared to the unimolecular structures. While DAP is hydrophilic in nature, it is not sufficient for achieving complete wetting of the surface of the Cu foam compared to the co-assembled structures. This emphasizes the need for surface roughness, as in the co-assembled structures, to achieve superhydrophilicity. Figures 7c and 7d display the SEM images of the Cu foam before and after coating with the co-assembled structures, respectively. From these images, it is clear that the structure adhered to the Cu foam, thereby changing the surface properties of the naturally hydrophobic Cu foam to superhydrophilic and further suggesting that this change in the nature of the surface is attributed to the structures.

**Figure 7:**
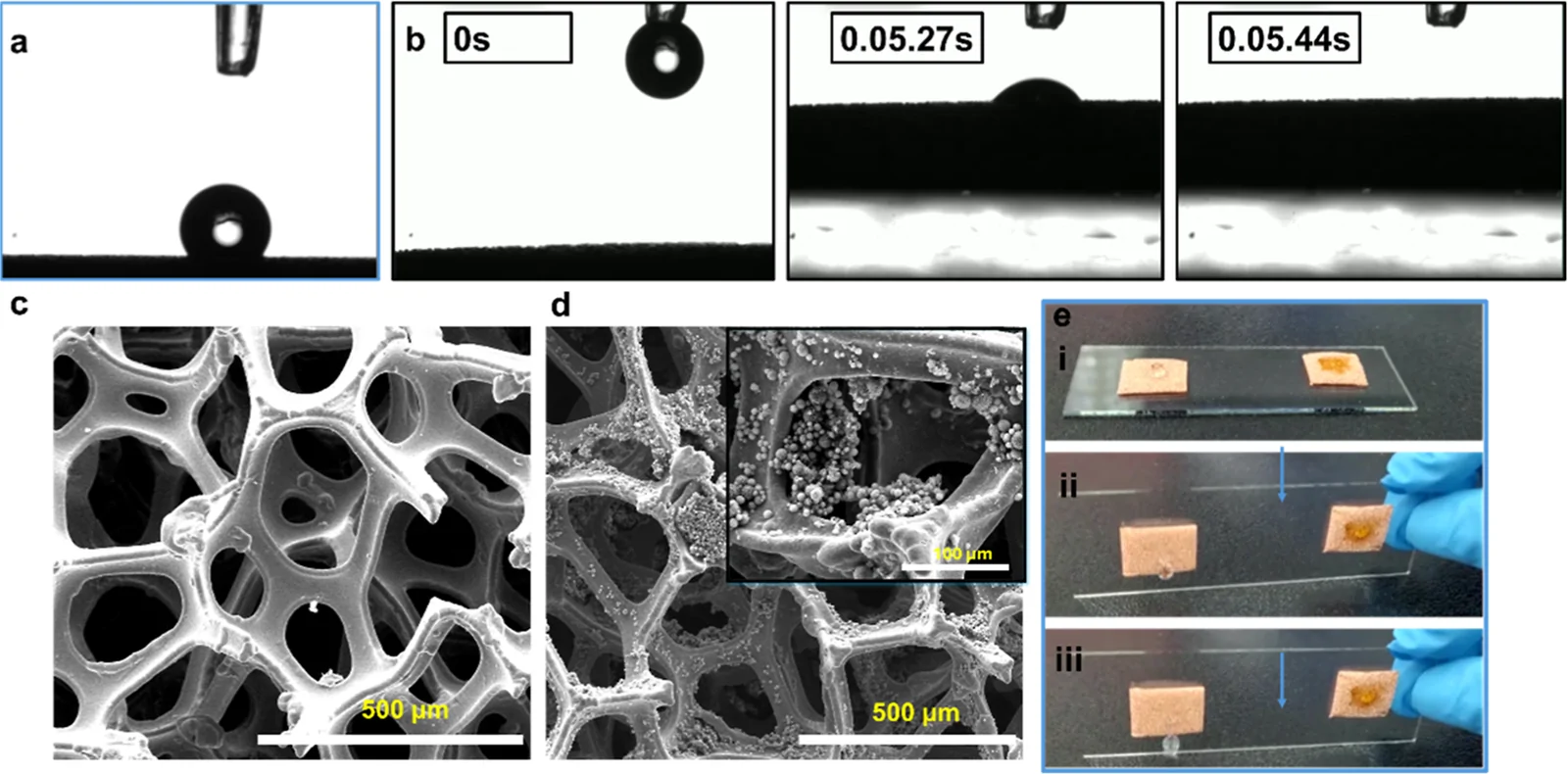
Photographs of the WCA of (a) Cu Foam, (b) Cu foam after coating with the co-assembled structures. The three consecutive photographs were taken before the water drop contacted the surface (*t* = 0 s) and immediately after the contact (*t* = 0.05.27 s). Complete spreading of the water drop occurred within 0.05.44 s (c) and (d) SEM images of the uncoated Cu Foam (c) and the coated Cu Foam (d). Inset SEM image of the coated Cu foam at higher magnification. (e) Three consecutive photographs taken from Video S1 of the two Cu foam surfaces: without (left) and with (right) coating of the Co-assembled structures. (i) Before tilting the samples. (ii) After tilting the samples. (iii) After tilting and tapping the samples.

Next, we aimed to demonstrate the ability of the fabricated hydrophilic–hydrophobic coated surface to adhere to water, compared to the uncoated hydrophobic Cu foam. For this purpose, we taped the two samples to a glass slide (Figure 7e,i) and drop-casted 10 μL of water on each. We then tilted the slide as shown in Figure 7e,ii. This caused the water to roll from the uncoated Cu foam. The drop on the coated Cu foam remained stable and showed strong adherence to the hydrophilic surface. Continuous tilting and tapping the slide (Figure 7e,iii, see Video S1) did not result in rolling of the drop from the coated Cu foam. This result demonstrates that this type of hydrophilic–hydrophobic coated surface can be used for further applications, such as water harvesting by decorating the Cu foam with patches of the co-assembled structures. The pronounced superhydrophilicity of the RF-DAP assemblies is likely a result of the combined contribution of surface chemistry and hierarchical morphology. While XPS revealed an increased surface abundance of nitrogen- and oxygen-containing groups in the RF-DAP structures, which can promote water interaction, the microspikes of the urchin-like architecture provide substantial surface roughness that can facilitate water spreading and capillary infiltration. The substrate-dependent measurements further support this combined effect. On glass, the water contact angle decreased from 57 ± 0.1° for bare glass to 0° after coating with RF-DAP assemblies. Similarly, coating hydrophobic Cu foam reduced the contact angle from 126 ± 0.1° to 0°, whereas DAP alone produced a contact angle of 52 ± 0.1°, and RF did not remain adhered to the Cu foam.

## 3 Conclusions

In summary, the coassembly of RF and DAP leads to the formation of urchin-like supramolecular structures, as confirmed by various analytical techniques including ITC, UV–vis, and FTIR. These structures exhibit stability, ITC measurements support entropy-favored association between RF and DAP under aqueous conditions; the relative contributions of these interactions to assembly under the preparation conditions remain to be established, with additional contribution from hydrogen bonds identified through FTIR analysis.

Moreover, the coassembly results in a new material with superhydrophilic properties, demonstrated by the enhanced hydrophilicity of glass coverslips upon coating with the co-assembled structures. This superhydrophilicity is attributed to both the presence of hydrophilic groups on the surface of the structures, as revealed by XPS experiments, and the surface roughness imparted by the microspikes of the urchin-like structures.

Importantly, this superhydrophilic property is not observed in the individual building blocks, RF or DAP, underscoring the significance of their coassembly in generating materials with unique properties. Additionally, the co-assembled structures were able to transform a superhydrophobic surface, such as Cu foam, into a superhydrophilic one, just by simply drop-casting the structures on the surfaces of the Cu foam, as was shown in the WCA. This enabled us to construct a hybrid surface combining hydrophilic–hydrophobic properties that can be used for future design of water or fog harvesting devices.

## 4 Experimental Section

### 4.1 Materials and Methods

Riboflavin and the nucleobases used in this study, 2,6-diaminopurine, adenine, cytosine, uracil, and thymine, were purchased from Sigma-Aldrich (purity >98%) and 1,1,1,3,3,3-hexafluoro-2-propanol (HFIP), 99% was purchased from Tzamal D-Chem.

### 4.2 Preparation of the Co-Assembled Structures

In a typical experiment, 2,6-diaminopurine powder (18.7 mg) was dissolved in a test tube using Milli-Q water to a concentration of 8.25 mM by vortexing and heating to 90 °C for 1 h until full dissolution. Riboflavin powder (12.5 mg) was dissolved in 1 mL HFIP to a final concentration of 33.21 mM. Next, riboflavin was added to the 2,6-diaminopurine solution in the following manner: For preparation of the RF-DAP 1:1 coassembly, 200 μL of the RF/HFIP solution was added to 800 μL of the DAP solution. For RF-DAP ratios of 1:2, 1:4, and 1:8, 100, 50, and 25 μL of the RF/HFIP solutions were used, respectively, while the DAP solution volume was maintained at 800 μL. The final volume of each sample was adjusted to 1 mL with Milli-Q water (100, 150, and 175 μL, respectively). Thus, the amount of DAP and the final sample volume were maintained constant throughout the series, while the amount of RF was varied. Unimolecular assemblies were prepared by adding riboflavin to a water solution with no 2,6-diaminopurine. For the control experiment using the nucleobases adenine, cytosine, uracil, and thymine, the same protocol was used except that 2,6-diaminopurine was replaced with the desired nucleobase at a 1:1 molar ratio. For the different characterization techniques, the co-assembled structures were spun down and washed with Milli-Q water to remove unreacted riboflavin and DAP. The precipitate was then redispersed in 200 μL of water.

### 4.3 Scanning Electron Microscopy (SEM)

A 7 μL aliquot of the relevant sample was allowed to dry on a microscope glass coverslip under ambient conditions overnight and was then coated with Au. SEM images were recorded using a Quanta 200 FEG (ThermoFisher) Environmental Scanning Electron Microscope (ESEM) operating at 10 kV in high vacuum mode.

### 4.4 Time-of-Flight Secondary Ion Mass Spectrometry (ToF-SIMS)

For ToF-SIMS analysis, the samples were deposited on a silicon chip (TED PELA, INC) and analyzed using a PHI model 2100 TRIFT II ToF-SIMS instrument. In this system, a pulsed primary ion beam was used to desorb and ionize species from the sample surface. The resulting secondary ions were accelerated into a mass spectrometer, where they were mass analyzed by measuring their time-of-flight from the sample surface to the detector. In addition, an image was generated by rostering a finely focused beam across the sample surface. Owing to the parallel detection nature of ToF-SIMS, the entire mass spectrum was acquired from every pixel in the image. The ions related to *m*/*z* 151.24 and *m*/*z* 377.44 were used to identify and evaluate the ionic image of 2,6-diaminopurine and riboflavin, respectively. The mass spectrum and the secondary ion images were then used to establish the composition and distribution of sample surface constituents.

### 4.5 Powder X-ray Diffraction (PXRD)

After sample preparation, the unimolecular components and the co-assembled structures were spun down, and excess water was drawn out. The co-assembled structures were washed with water 3 times to remove the unreacted components. Next, the samples were allowed to completely dry under vacuum. The self-or co-assembled structure powders were deposited on a quartz zero-background sample holder. PXRD patterns were collected using a Bruker D8 Discover diffractometer (Bruker, Germany) equipped with Goebels mirrors to parallelize the beam and a LYNXEYE-XE linear detector. Data collection was performed at room temperature with a scan range of 2θ 5–40°.

### 4.6 Isothermal Titration Calorimetry (ITC)

2,6-Diaminopurine (40 mM) was dissolved in 1x PBS, and riboflavin was diluted in PBS to a working concentration of 1 mM. All titration experiments were performed at 25 °C in 1× PBS buffer (pH 7.2). The instrument was equilibrated at 25 °C until the baseline was flat and stable. The stirring speed was maintained at 700 rpm during the titrations. The solutions were kept at 25 °C and incubated at that temperature for 5 min before measurement. A 350 μL riboflavin sample was inserted into the Nano ITC low-volume cell (Malvern MicroCal PEAQ-ITC), and the titrating syringe was filled with 50 μL DAP solution. The excess solution was removed from the cell before performing the experiments. The system was allowed to reach a stable temperature of 25 °C for 600 s, and baseline data were collected for 120 s. Subsequently, DAP was titrated into PBS as a control and subtracted from the final measurements. Titration was carried out in 2 μL aliquots and allowed to equilibrate for 180 s before the next drop, with a total of 20 drops, a total of 38.5 μL (the first drop was half volume). The resulting isotherm was analyzed using the Malvern MicroCal PEAQ-ITC Analysis Software using an independent interaction model. The binding isotherm was fitted to a “one set of binding sites” model.

### 4.7 Fourier Transform Infrared (FTIR) Spectroscopy

Transmission infrared spectra were collected using a nitrogen-purged Nexus 470 FTIR spectrometer (Nicolet, Offenbach, Germany) equipped with a deuterated triglycine sulfate (DTGS) detector. Measurements were performed by averaging 64 scans at 4 cm^–1^ resolution. To obtain the FTIR spectra, 20 μL of the sample was deposited on a disposable KBr infrared sample card (Sigma-Aldrich, Rehovot, Israel), which was then allowed to dry under vacuum.

### 4.8 UV–Vis Spectra

UV–vis spectra of the samples were recorded using a T60 UV–vis spectrophotometer (PG Instruments, Leicestershire, UK). A quartz cuvette (Hellma Analytics, Müllheim, Germany) with an optical path length of 0.01 mm was used.

### 4.9 Water Contact Angle (WCA)

The wettability states of uncoated or coated glass coverslip/micro porous Cu foam were estimated by measuring the WCA of sessile drops of deionized water (pH, 5.5; resistivity >17 MΩ cm) placed on a sample surface. Measurements were performed using a ramé-hart model 400 Goniometer with a 520 frames/second SuperSpeed digital camera and DROPimage Standard Software. The liquid volume was kept constant (0.25 μL) for all contact angle measurements of different specimens. The data for each measurement were recorded as the mean value of the angle measurements obtained from the two sides of each water droplet. For superhydrophilic co-assembled coated substrates, a movie was recorded to capture the wetting dynamics and the images were processed using the Microsoft Clipchamp software. Contact angles were calculated using custom-made computer software that fits a solution of the Young–Laplace equation to the measured radius of the base of the drop, given its volume, surface energy and density. The wettability investigations were carried out with an accuracy of ±0.1°.

### 4.10 X-ray Photoelectron Spectroscopy (XPS)

The samples were deposited on a silicon chip and were allowed to dry overnight under vacuum. XPS measurements were performed using an ESCALAB QXi (Thermo Scientific, USA) system. The samples were irradiated with monochromatic Al Kα radiation. High-resolution spectra of C 1s, N 1s, O 1s, F 1s and Si 2p were collected with a pass energy of 20 eV and a 400 μm spot size. Samples were cleaned using an Ar cluster ion beam with an energy of 6 kV and a minimum cluster size of 300.

### 4.11 Fabrication of Amphiphilic Surface

The co-assembled structures, dispersed in water, were deposited on a hydrophobic surface of Cu foam and allowed to dry overnight under vacuum. Next, the coated Cu-foam was dried with nitrogen to ensure that residual water was removed from the surface. For images shown in Figure 7e, the coated and uncoated Cu foams were taped to a glass microscope slide using carbon tape. The glass slide was tilted and tapped to demonstrate the adhesion of a water drop to the coated surface.

## Supplementary Material





## Notes

SEM image of RF and DAP coassemblies at a 1:2 molar ratio (Figure S1); images of the visual appearance of RF solutions upon mixing with nucleobases A, C, T, U, and DAP at a 1:1 ratio (Figure S2); thermodynamic values of RF and DAP binding as assessed by ITC (Table S1); photographs of the WCA of Cu foam after coating with DAP only (Figure S3); Water-adhesion test of uncoated (left) and RF-DAP coassembly coated (right) Cu foam taped to a glass slide. A 10 μL water droplet was drop-casted on each sample, and the slide was then tilted and tapped. The droplet rolls off the uncoated, hydrophobic Cu foam, whereas the droplet on the coated, superhydrophilic Cu foam remains in place and does not roll off, even after continued tilting and tapping (PDF)
Video of the water-adhesion test of uncoated and RF-DAP-coated Cu foam upon tilting and tapping (Video S1); File name: Video_S1.mp4 (MP4)
