# Supplementary material for: Bio-Inspired Superhydrophilic Urchin-Like Supramolecular Structures Formed by Co-Assembly of Simple Metabolites
Source: Langmuir. 2026 Oct 20;42(43):31727–36. doi: 10.1021/acs.langmuir.6c03644 (PMC13738784; doi:10.1021/acs.langmuir.6c03644)
Supplement: Supplementary file 1 [file la6c03644_si_001.pdf]

Supporting Information

Bio-inspired Superhydrophilic Urchin-like Supramolecular Structures formed by Co-assembly of Simple Metabolites

Ruth Aizen[1], Om Shanker Tiwari[2], Gal Finkelstein-Zuta[1,4], Pini Shekhter[3], Davide Levy[3], Ehud Gazit[1,4*]

1. The Shmunis School of Biomedicine and Cancer Research, The George S. Wise Faculty of Life Sciences, Tel Aviv University, Tel Aviv 6997801, Israel.
2. Department of Chemistry, National Institute of Technology Warangal, Hanamkonda 506004, Telangana, India.
3. Jan Koum Center for Nanoscience and Nanotechnology, Tel Aviv University, Tel Aviv 6997801, Israel.
4. Department of Materials Science and Engineering, The Iby and Aladar Fleischman Faculty of Engineering, Tel Aviv University, Tel Aviv 6997801, Israel.

Author Information

*Corresponding Author

Address: The Shmunis School of Biomedicine and Cancer Research, The George S. Wise Faculty of Life Sciences, Tel Aviv University, Tel Aviv 6997801, Israel.

Email: ehudg@post.tau.ac.il (Prof. Ehud Gazit)

Table of Contents

	Page No
SEM image of RF and DAP co-assemblies at 1:2	**S-2**
Images of the visual appearance of the solutions of RF upon mixing with nucleobases	**S-2**
Thermodynamic values of RF and DAP binding as assessed using ITC measurements	**S-3**
Photographs of the WCA of Cu foam after coating with DAP only	**S-3**
Water-adhesion test of uncoated and RF-DAP-coated Cu foam	**S-3**

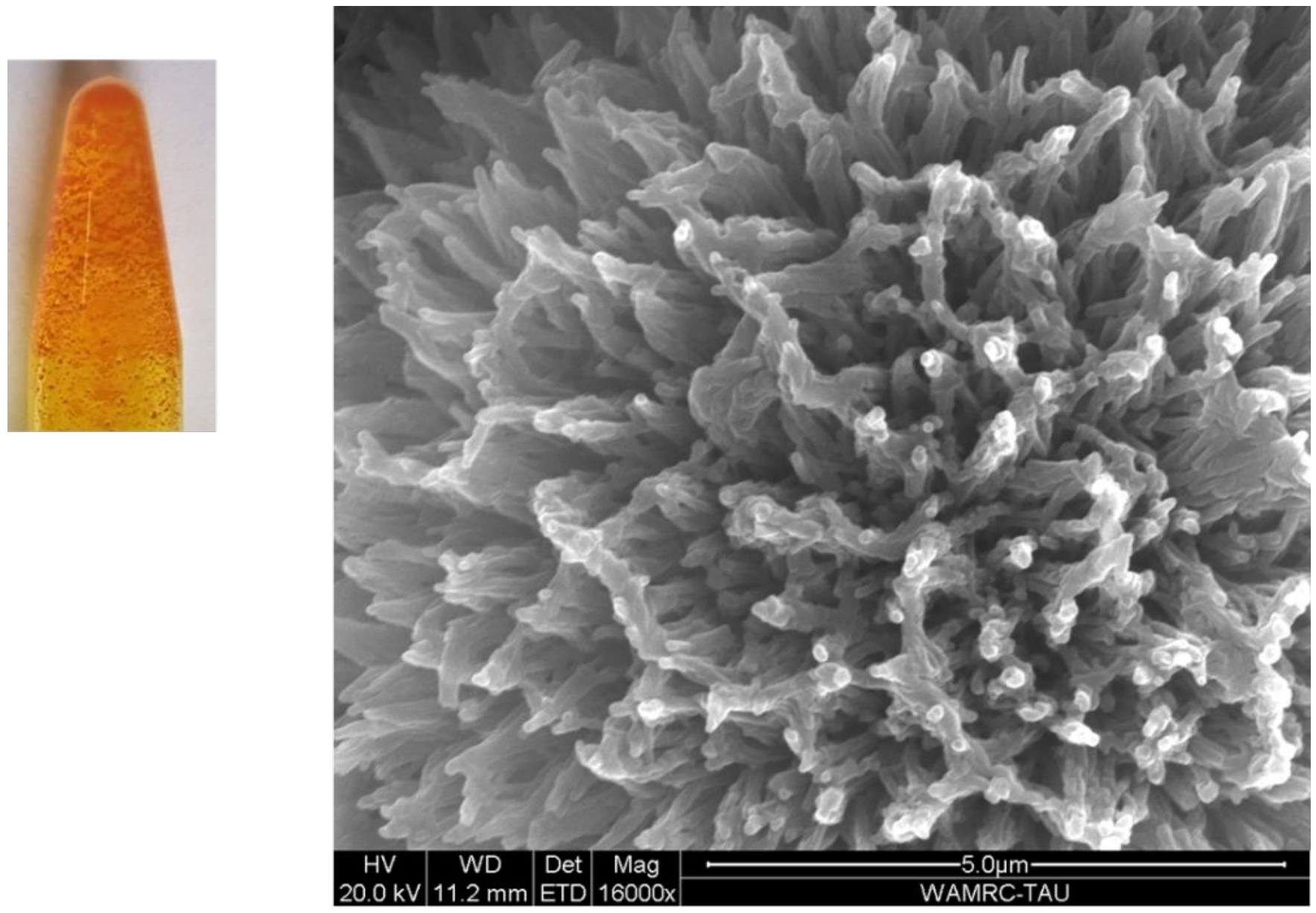

Figure S1. SEM image of RF and DAP co-assemblies at 1:2.

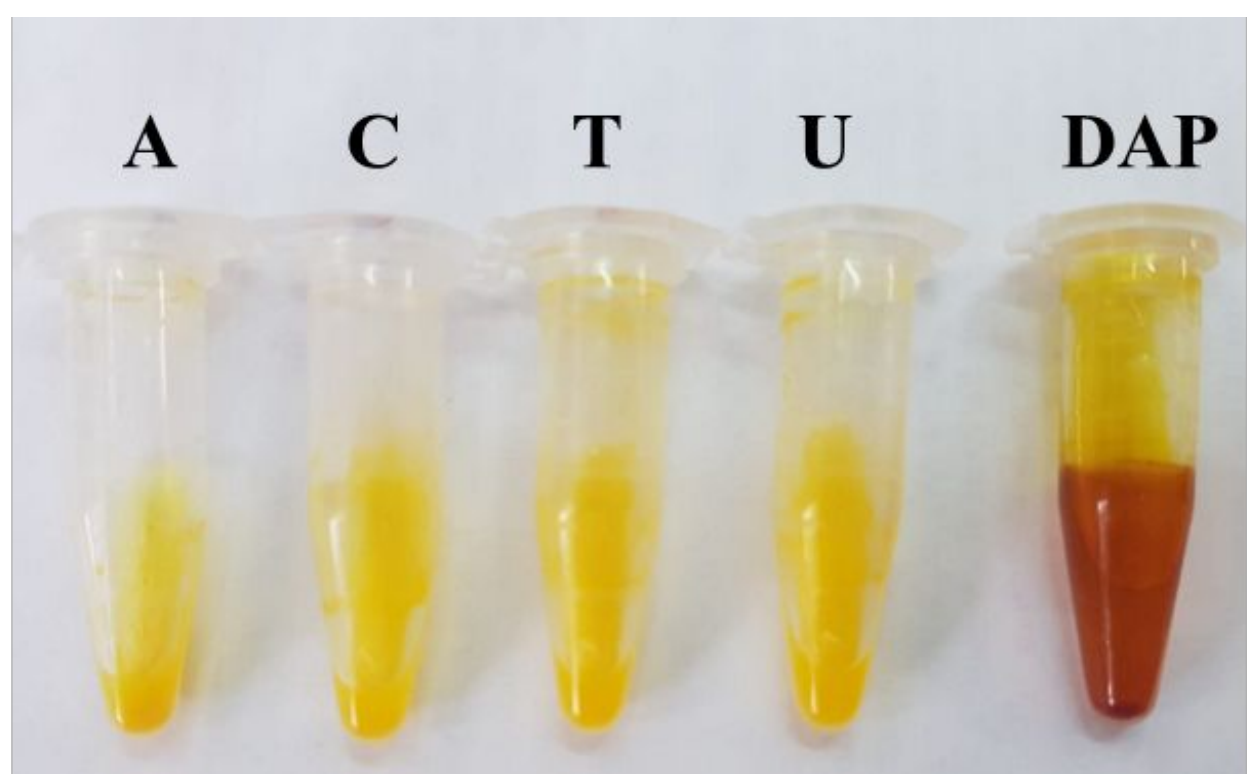

Figure S2. Images of the visual appearance of RF solutions upon mixing with nucleobases A, C, T, U, and DAP at a 1:1 ratio, as indicated.

Table S1. Thermodynamic values of RF and DAP binding as assessed using ITC measurements.

**Metabolites**	**Thermodynamic** Parameter	**Standard Deviation**
RF and DAP	N = 1.64<br>K_D (M) = 27.1 x 10^{-6},<br>ΔH (kJ/mol) = 1.38,<br>ΔG (kJ/mol) = -26.1,<br>-TΔS (kJ/mol) = -27.5,	± 0.025<br>± 6.09 x 10^{-6},<br>± 0.033,<br>---------<br>---------

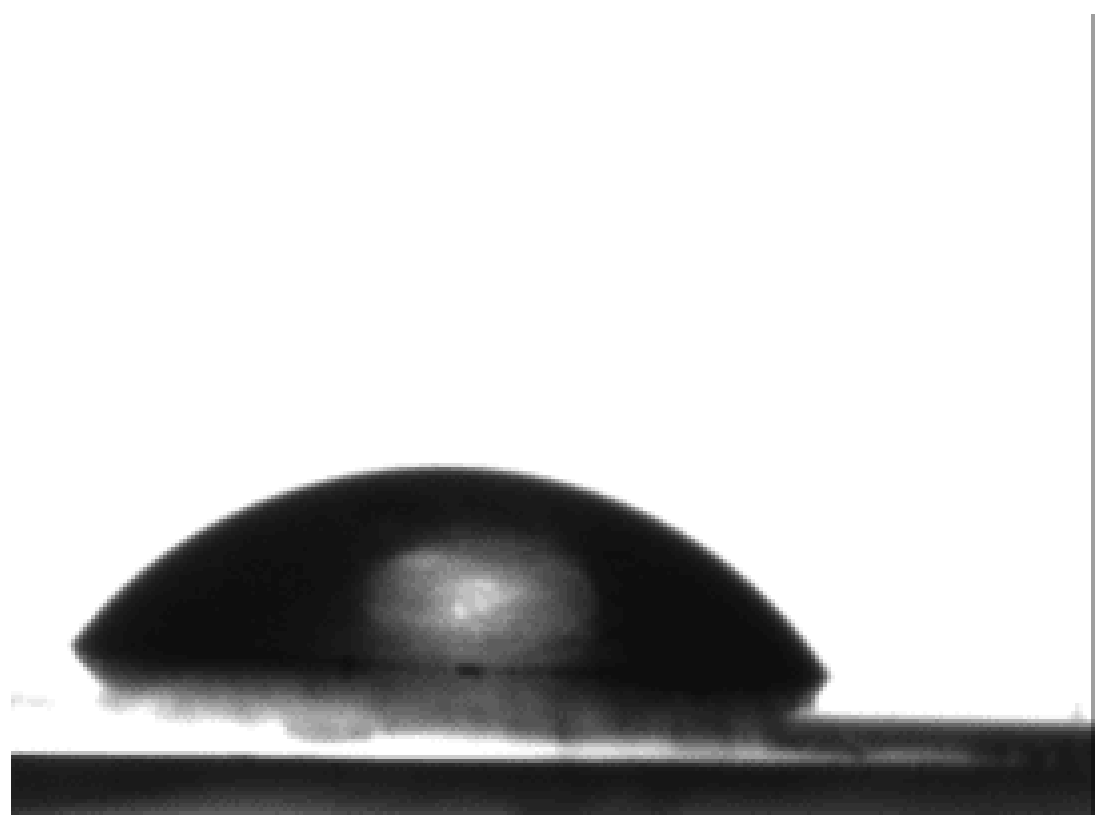

Figure S3. Photographs of the WCA of Cu foam after coating with DAP only. WCA was measured to be θ=52 ± 0.1°.

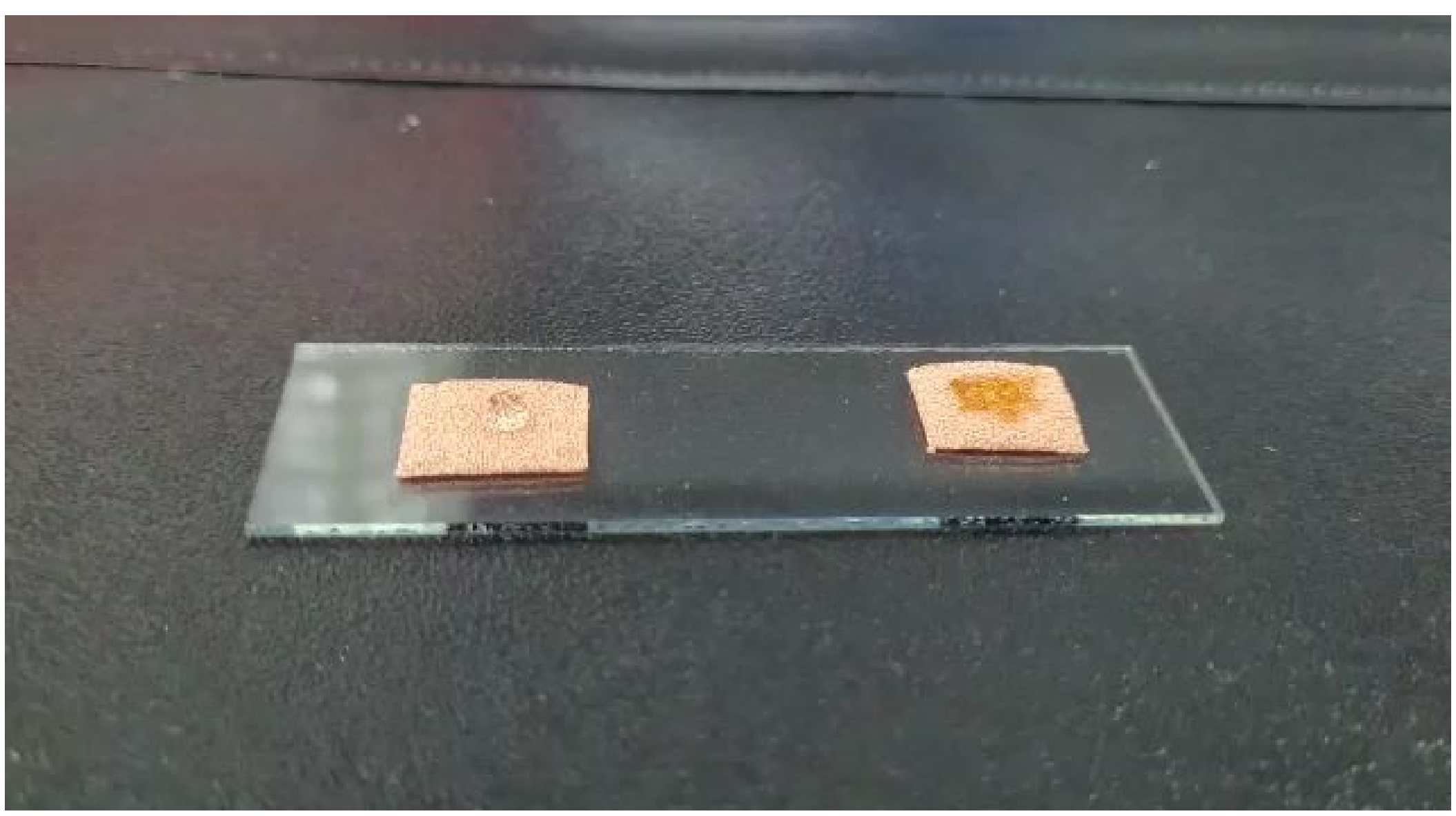

Video S1. Water-adhesion test of uncoated (left) and RF-DAP co-assembly-coated (right) Cu foam taped to a glass slide. A 10 µL water droplet was drop-casted on each sample, and the slide was then tilted and tapped. The droplet rolls off the uncoated, hydrophobic Cu foam, whereas the droplet on the coated, superhydrophilic Cu foam remains in place and does not roll off, even after continued tilting and tapping. File name: Video_S1.mp4
